# Metagenomes and metatranscriptomes from the L4 long-term coastal monitoring station in the Western English Channel

**DOI:** 10.4056/sigs.1202536

**Published:** 2010-10-27

**Authors:** Jack A. Gilbert, Folker Meyer, Lynn Schriml, Ian R Joint, Martin Mühling, Dawn Field

**Affiliations:** 1 Plymouth Marine Laboratory, Prospect Place, Plymouth, PL1 3DH, U.; 2 Argonne National laboratory, 9700 S. Cass Ave., Argonne, IL 60439, USA; 3 University of Chicago, Chicago, IL 60637, USA; 4 University of Maryland School of Medicine, 655 W. Baltimore Street, Baltimore MD 21201; 5 TU Bergakademie Freiberg, IÖZ - Interdisciplinary Centre for Ecology, Leipziger Str. 29, 09599 Freiberg, Germany.; 6 NERC Centre for Ecology and Hydrology, Mansfield Road, Oxford, OX1 3SR, UK

**Keywords:** Marine, aerobic, surface water, coastal, temperate, metagenome, metatranscriptome, pyrosequencing, time-series, diel, seasonal

## Abstract

Both metagenomic data and metatranscriptomic data were collected from surface water (0-2m) of the L4 sampling station (50.2518 N, 4.2089 W), which is part of the Western Channel Observatory long-term coastal-marine monitoring station. We previously generated from this area a six-year time series of 16S rRNA V6 data, which demonstrated robust seasonal structure for the bacterial community, with diversity correlated with day length. Here we describe the features of these metagenomes and metatranscriptomes. We generated 8 metagenomes (4.5 million sequences, 1.9 Gbp, average read-length 350 bp) and 7 metatranscriptomes (392,632 putative mRNA-derived sequences, 159 Mbp, average read-length 272 bp) for eight time-points sampled in 2008. These time points represent three seasons (winter, spring, and summer) and include both day and night samples. These data demonstrate the major differences between genetic potential and actuality, whereby genomes follow general seasonal trends yet with surprisingly little change in the functional potential over time; transcripts tended to be far more structured by changes occurring between day and night.

## Introduction

The Western Channel Observatory station L4, located off the Plymouth coast in the UK, has been collecting environmental data for almost a century [1]. This includes published 16S rRNA V6 amplicon pyrosequencing data cataloging monthly patterns in microbial diversity [2,3]. The importance of the area rests with its being a transition zone between many northern and southern planktonic species [1] and with the fact that, as a major confluence between the North Atlantic Ocean and the North Sea, water masses exhibit extremely short residence times (>2 months [4]; ). In the study reported here, we use shotgun metagenomics and metatranscriptomics to identify the relationship between genetic and functional diversity at station L4.

## Classification and features

### Relationship of reported datasets

**Figure 1:**
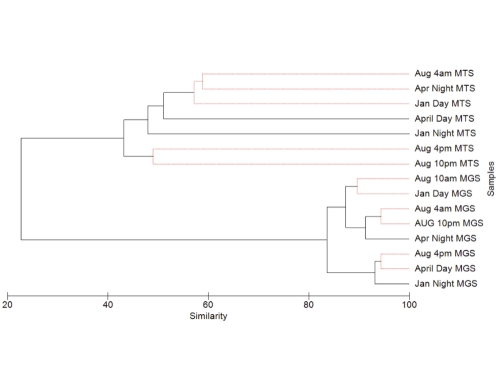
Group-average dendrogram showing relationship between all metagenomes and metatranscriptomes, based on comparison of annotated protein fragments via BLAST x using the SEED database in MG-RAST for each dataset. MTS – metatranscriptome. MGS – metagenome.

We generated 8 metagenomes and 7 metatranscriptomes for eight time points. Figure 1 shows the relationships of these metagenomes and metatranscriptomes; the figure was produced by using a group-average clustering dendrogram representing the relationships based on comparison of 66,529 amino acid sequences of greater than 40 amino acids predicted from each dataset (for details of the process, see Metagenome Annotation). One can clearly see that the metagenomic and metatranscriptomic data cluster separately. The metagenomic data shows an average similarity of less than 2%, clustered by season, from which one can infer that the seasonal differences are stronger than the diel differences. On the other hand, the metatranscriptomes show more similarity and a tendency to cluster by diel time point; specifically, the April night data and January night data are more similar to each other than either is to the April day data and January day data. The August metatranscriptomes cluster by themselves, but this clustering is also structured by day and night. Table 1 details the classification and general features of the metagenomic datasets information for this study in MIMS format.

**Table 1:** Classification and general feature of 8 metagenome datasets according to the MIMS recommendations [5].

**MIGS ID**	**Property**	**Term**	**Evidence code**
	Current classification	Metagenome ecological metagenome marine metagenome	TAS [6]
5	Collection date	Jan Day: 2008-01-28T15:30 Jan Night: 2008-01-28T19:00 Apr Day: 2008-04-22T16:00 Apr Night: 2008-04-22T22:00 Aug 4pm: 2008-08-27T16:00 Aug 10 pm: 2008-08-27T22:00 Aug 4 am: 2008-08-28T04:00 Aug 10 am: 2008-08-28T10:00	TAS [6]
6	Latitude Longitude	Jan Day: 50.2518:4.2089 Jan Night: 50.2611:4.2435 Apr Day: 50.2518:4.2089 Apr Night: 50.2530:4.1875 Aug 4pm: 50.2518:4.2089 Aug 10 pm: 50.2545:4.1990 Aug 4 am: 50.2678:4.1990 Aug 10 am: 50.2665:4.1486	NAS
7	Depth	0	NAS
8	Altitude	0	NAS
9	Geographic location/Country	England	NAS
10	Environment	Coastal Marine	
11a	Environmental Package	See Table 2	
29	Sample collection device or method	Large bore peristaltic filtration pump	
30	Sample material processing	Water filtered on to a 0.22 µm Sterivex (Millipore) filter and then snap-frozen at -80C	
31	Amount or size of sample collected	10L	

Evidence codes - IDA: Inferred from Direct Assay (first time in publication); TAS: Traceable Author Statement (i.e., a direct report exists in the literature); NAS: Non-traceable Author Statement (i.e., not directly observed for the living, isolated sample, but based on a generally accepted property for the species, or anecdotal evidence). These evidence codes are from the Gene Ontology project [7]. If the evidence code is IDA, then the property was directly observed for a live isolate by one of the authors or an expert mentioned in the acknowledgements.

### Environmental characteristics and descriptions

Environmental data was collected for temperature, density, salinity, chlorophyll a, total concentration of organic nitrogen and carbon, nitrate, ammonia, silicate, and phosphate [Table 2]. The methods used are described on the Western Channel Observatory website.

**Table 2:** Environmental variables for each sampling occasion

**Property**	**Measurement^a^**								
Sample Collection date (MIGS-5)	**01/28**	**01/28**	**04/22**	**04/22**	**08/26**	**08/26**	**08/27**	**08/27**	**Evidence code**
Sample collection time	15:38	19:30	16:00	22:00	16:00	22.00	04:00	10:00	
Temperature (ºC)	10.1	10.1	9.7	9.6	15.9	15.8	15.7	15.8	IDA
Density (kg m^-2^)	1025.6	1026.3	1027.2	1027.1	1023.5	1024.3	1024.5	1024.4	
Salinity (PSU)	33.3	34.2	35.1	35.0	32.1	33.0	33.3	33.2	
Chlorophyll a (µg/L)	0.8	0.9	2.2	1.3	9.2	8.2	9.8	11.9	IDA
Total Organic Nitrogen (µmol L-1)	1.3	3.5	2.9	2.8	2.8	2.3	3.0	4.1	IDA
Total Organic Carbon (µmol L-1)	33.2	38.2	27.2	19.4	26.8	26.5	22.0	23.7	IDA
NO2 + NO3 (µmol L-1)	10.9	10.0	4.0	3.8	0.1	0.1	0.9	0.1	
Ammonia (µmol L-1)	0.0	0.0	0.5	0.3	0.1	0.1	0.1	0.1	IDA
SRP (µmol L-1)	0.5	0.5	0.4	0.3	0.0	0.1	0.0	0.1	
Silicate (µmol L-1)	6.0	5.8	2.6	2.7	0.1	0.2	0.3	0.2	

^a^ Samples collected January – August, 2008. Evidence codes: MIGS-5: TAS [5].

**Figure 2:**
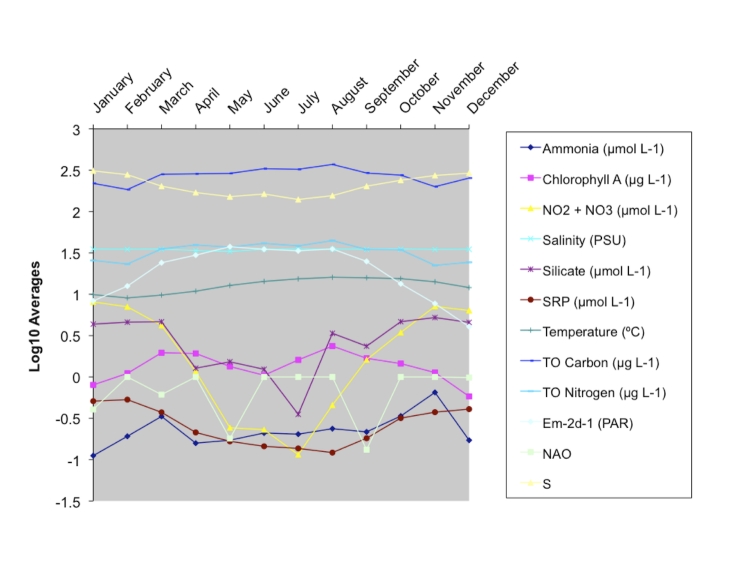
Monthly annual averages for all environmental parameters and species richness (S). TO – total organic; SRP – Soluble Reactive Phosphorous; PAR – Photosynthetically Active Radiation; NAO – North Atlantic Oscillation. Data taken from Gilbert et al., 2010.

**Figure 3:**
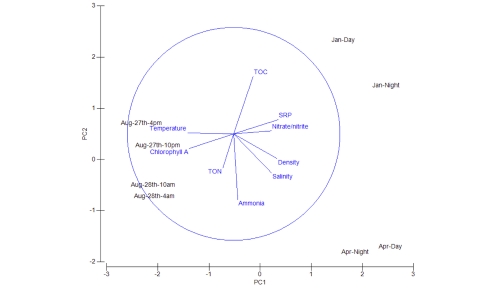
Principal component analysis of environmental variables showing the seasonal differences in variables outlined in Table 2. Classification and general features of the 15 datasets in accordance with the MIMS recommendations [5]

(http://www.westernchannelobservatory.org.uk/all_parameters.html).Figure 2 plots the environmental trends at L4 averaged for the years 2003-2008; the graph clearly shows the differences among the samples taken in the three months. Figure 3 shows a principal component analysis of the environmental parameters recorded during this study. Evident from the figure is the fact that the January samples have higher nutrient concentrations, the April samples show changes in the water salinity as a consequence of density, and the August samples show changes in temperature and chlorophyll a concentration.

## Metagenome sequencing and annotation

### Metagenome project history

Two factors motivated the choice of station L4: its century-long history of environmental data [8] and the six years of 16S rRNA V6 amplicon pyrosequencing information detailing microbial diversity patterns [2,3], from which we inferred interannual variability from our single-year study. All 16S rRNA V6 amplicon pyrosequencing data have been submitted to the NCBI short reads archive under SRA009436 and registered with the GOLD database (Gm00104). The data also can be accessed from the VAMPS server (http://vamps.mbl.edu/index.php). The metagenomic data and metatranscriptomic data are available on the CAMERA website under Western Channel Observatory Microbial Metagenomic Study (http://web.camera.calit2.net) and on the Metagenome Rapid Annotation using Subsystem Technology (MG-RAST) system under 4443360-63, 4443365-68 and 4444077, 4445065-68, 4445070, 4445081, and 4444083 (http://metagenomics.nmpdr.org/), as well as through the INSDC short-reads archive under ERP000118 (http://www.ebi.ac.uk/ena/data/view/ERP0001180). Table 1, Table 2, Table 3, and Table 4 detail the metagenomic sequencing project information for this study in MIMS format.

**Table 3:** Metagenome sequencing project information (MIMS compliance)

MIGS ID	Property	**Jan 3pm**	**Jan 7pm**	**Apr 4pm**	**Apr 10pm**	**Aug 4pm**	**Aug 10pm**	**Aug 4am**	**Aug 10am**
**35**	**library reads sequenced**	616,793	784,823	637,801	493,003	620,759	524,953	500,117	326,475
**32**	**nucleic acid extraction**	Gilbert et al. 2008							
**43**	**sequencing method**	454 Titanium pyrosequencing (GS flx)							
**46**	**Assembly**	none							
	**INSDC ID**	SRA009436							
	**GenBank Date of Release**	01-12-2009							
	**GOLD ID**	GM00104							

**Table 4:** Metatranscriptome sequencing project information (MIMS compliance)

**MIGS ID**	**Property**	**Jan 3pm**	**Jan 7pm**	**Apr 4pm**	**Apr 10pm**	**Aug 4pm**	**Aug 10pm**	**Aug 4am**
**35**	**library reads sequenced**	139,880	130,826	124,925	147,492	139,375	193,254	154,865
**32**	**nucleic acid extraction**	Gilbert et al. 2008						
**43**	**sequencing method**	454 Titanium pyrosequencing (GS flx)						
**46**	**Assembly**	none						
	**INSDC ID**	SRA009436						
	**GenBank Date of Release**	01-12-2009						
	**GOLD ID**	GM00104						

### Sampling and DNA isolation

For the sampling, a minimal-impact surface buoy was deployed with a 7 m current drogue following a Lagrangian drift. Samples were taken at station L4 to represent three seasons and both day and night readings, as follows:

- Winter: January 28, at 3:00 pm and again at 7 pm (2 hours after sundown) at 50.2611 N: 4.2435 W
- Spring: April 22, at 4 pm and again at 10 pm (one and a half hours after sundown) at 50.253N:4.1875W
- Summer: August 27, at 4 pm and again at 10 pm (two hours after sundown) at 50.2545N:4.199W
- Summer: August 28, at 4 am (two hours before sunrise) at 50.2678N:4.1723W and at 10 am at 50.2665N:4.1486W

The sampling technique involved the following steps: (1) collection of 20 L of seawater from the surface (0-2 m), (2) prefiltering through a 1.6 µm GF/A filter (Whatmann), (3) passage of the filtrate through a 0.22 µm Sterivex cartridge (Millipore) for a maximum of 30 minutes (approximately 10 L per Sterivex cartridge); (4) pump-drying and snap-freezing of the cartridges in liquid nitrogen, (5) barcoding [9] of the samples at the laboratory, and (6) storage at -80 °C.

Both DNA and RNA then were isolated from each sample [2,10], barcoded, and stored at -80°C. DNA and mRNA-enriched cDNA were purified from the samples; for details, see [10].

### Metagenome sequencing and assembly

The isolated DNA was used for metagenomic analysis, and the mRNA-enriched cDNA was used for metatranscriptomic pyrosequencing analysis. All DNA and cDNA were pyrosequenced on the GS-FLX Titanium platform. No DNA assembly was carried out.

### Metagenome annotation

The MG-RAST bioinformatics server [11] was used for annotating the metagenomic samples [1-6,8-14]. The data also were processed by using custom-written programming scripts on the Bio-Linux system [6] at the NERC Environmental Bioinformatics Centre (http://nebc.nerc.ac.uk/tools/scripts) unless otherwise indicated. In order to ensure high quality, the following sequences were removed from the pyrosequenced data: transcript fragments with >10% non-determined base pairs (Ns), fragments <75 bp in length, fragments with >60% of any single base, and exact duplicates (resulting from aberrant dual reads during sequence analysis). So-called artificial duplicates in the metagenomic data (i.e., multiple reads that start at the same position; see, e.g., Gomez-Alvarez et al., 2009) were not removed, however, because of the possibility of their being natural; their removal would have precluded comparison with the metatranscriptomic data [13].

The nucleic acid sequences were then compared with three major ribosomal RNA databases – (SILVA (http://www.arb-silva.de/), RDP II (http://rdp.cme.msu.edu/), and Greengenes (http://greengenes.lbl.gov) – using the bacterial and archaeal 5S, 16S, and 23S and the eukaryotic 18S and 25S sequence annotator function of MG-RAST (e-value < 1 x 10-5; minimum length of alignment of 50 bp; minimum sequence nucleotide identity of 50%). Reads annotated as rRNA were excluded. All subsequent reads were considered to be valid DNA or valid putative mRNA derived sequences and were annotated against the SEED database using MG-RAST (e-value < 1 x 10-3; minimum length of alignment of 50 bp; minimum sequence nucleotide identity of 50%; Meyer et al., 2008). The result was an abundance matrix of functional genes and protein-derived predicted taxonomies across the DNA and mRNA samples.

The sequences also were translated using the techniques described by Gilbert *et al*. (2008) and Rusch *et al.* (2007) [10,14]. Predicted open reading frames (pORFs) having >40 amino acids were produced in all six reading frames. The CD-HIT program [15] was used to cluster the proteins from the datasets at 95% amino acid identity over 80% of the length of the longest sequence in a cluster. The longest representative from each cluster then was clustered at 60% amino acid identity over 80% of the length of the longest sequence to group these sequences by protein families. Based on the relative abundance of each sample in a cluster, an abundance matrix was created using the output cluster files from CD-HIT that contained the original fasta sequences and headers for each sample (*abundanceMatrix-twoStep.pl).* Subsequently, protein clusters with ≤2 representative pORFs were removed from the pORF matrix (*MatrixParser.pv*). In order to equalize the sequencing effort, all samples were randomly resampled (*Daisychopper.pl*) to the same number of pORFs or sequences across the clusters or functional/taxonomic SEED annotations.

## Metagenome properties

Approximately 4.5 million combined microbial metagenomic reads were produced, comprising ~1.9 billion bp, with an average read length of ~350 bp across the eight samples, ranging from 326,475 to 784,823 sequences [Table 5]. Seven metatranscriptomic datasets were also produced (the sample taken on August 28 at 10 am was lost in transit) totaling ~1 million sequences. After cleanup, 392,632 putative mRNA-derived sequences remained, totaling 159 million bp, with an average of 272 bp per sequence. The effort per sample varied from 33,149 to 96,026 sequences [Table 6]. SEED annotations produced via MG-RAST (Table 7 and Table 8 ranged from 20% to 46% of each metagenomic dataset and from to 11% to 35% of the metatranscriptomic datasets.

**Table 5:** Metagenome statistics

	**Jan 3pm**	**Jan 7pm**	**Apr 4pm**	**Apr 10pm**	**Aug 27 4pm**	**Aug 27 10pm**	**Aug 28 4am**	**Aug 28 10am**
No. Original DNA Sequences	616,793	784,823	637,801	493,003	620,759	524,953	500,117	326,475
Predicted ORFs (>40aa pORFs)	862,695	1,287,412	1,003,799	745,305	986,269	846,209	779,951	491,330
No. of pORF clusters (95%)	615,374	1,123,829	779,342	588,387	881,113	703,712	675,210	444,729
No. of pORF singletons (95%)	546,463	1,031,865	682,586	526,233	805,284	634,042	608,785	410,616
No. of pORF ‘families’ (60%)	423,674	1,031,904	678,547	528,213	801,760	637,542	620,403	419,461
No. of pORF singletons (60%)	352,938	962,073	609,351	486,712	740,032	589,839	577,027	398,202
**Resampled pORFs (66529)**								
No. of pORF clusters (95%) (66529)	56337	64446	61187	59904	65601	63032	64729	65075
No. of pORF singletons (95%) (66529)	52891	63378	58691	57779	64818	61068	63359	63945
Good’s Coverage (66529)	20.5	4.7	11.8	13.2	2.6	8.2	4.8	3.9
No. DNA seqs with functional annotation	122,936	291,953	258,658	164,249	283,761	196,369	196,972	126,392
No. DNA seqs without functional annotation (%)	493,857	492,870	379,143	328,754	336,998	328,584	303,145	200,083
Percent DNA seqs without functional annotation	80%	63%	59%	67%	54%	63%	61%	61%
No. DNA seqs with taxonomic annotation	190,326	417,920	349,888	241,541	379,911	288,356	304,003	186,421
**Resampled sequencing effort (186,421)**								
Number of archaeal sequences (186,421)	19,055	15,150	777	561	1,370	1,093	1,585	1,244
Number of bacterial sequences (186,421)	161,899	146,911	182,850	180,674	182,717	176,825	180,725	182,332

**Table 6:** Metatranscriptome statistics

	Jan 3pm	Jan 7pm	Apr 4pm	Apr 10pm	Aug 27 4pm	Aug 27 10pm	Aug 28 4am
No. Original cDNA Sequences	139,880	130,826	124,925	147,492	139,375	193,254	154,865
No. of sequences following filtering***	94,024	106,864	84,916	109,577	87,799	118,360	111,568
No. mRNA following removal of rRNA	61,831	96,026	41,378	53,413	33,149	51,829	55,006
Predicted ORFs (>40aa pORFs)	143,169	211,374	81,642	107,699	77,985	66,529	159,909
No. of pORF clusters (95%)	98,871	78,278	35,648	51,088	28,167	24,136	68,080
No. of pORF singletons (95%)	82,464	54,870	25,925	38,960	19,600	17,177	50,246
No. of pORF ‘families’ (60%)	84,598	45,049	19,131	37,628	15,146	12,735	41,480
No. of pORF singletons (60%)	76,655	30,720	13,869	30,919	9,857	9,134	32,662
**Resampled pORFs (66529)**							
No. of pORF clusters (95%) (66529)	31026	50354	30334	34217	24848	24136	33191
No. of pORF singletons (95%) (66529)	23038	43687	22394	26840	17373	17177	25636
Good’s Coverage (66529)	65.37	34.33	66.34	59.66	73.89	74.18	61.47
No. mRNA seqs with functional annotation	11,513	31,990	8,845	16,315	11,720	5,907	15,384
No. mRNA seqs without functional annotation	50,318	64,036	32,533	37,098	21,429	45,922	39,622
Percent DNA seqs without functional annotation	81%	67%	79%	69%	65%	89%	72%
No. mRNA seqs with taxonomic annotation	29,521	30,778	20,899	26,398	15,456	29,605	38,304
**Resampled sequencing effort (15,456)**							
Number of archaeal sequences (15,456)	625	49	1	16	4	4	11
Number of bacterial sequences (15,456)	13,633	11,926	13,702	8,449	14,469	15,071	14,803

**Table 7:** Number of genes associated with the general SEED functional categories

**Subsystem Hierarchy 1**	**Jan 3pm**	**Jan 7pm**	**April 4pm**	**April 10pm**	**Aug 27 4pm**	**Aug 27 10pm**	**Aug 28 4am**	**Aug 28 10am**
Amino Acids and Derivatives	13,515	12,346	13,913	12,089	13,279	12,517	11,966	12,074
Carbohydrates	14,181	13,087	14,884	13,829	14,801	13,929	13,258	13,780
Cell Division and Cell Cycle	2,136	2,026	2,286	2,243	2,243	2,231	2,175	2,234
Cell Wall and Capsule	5,632	5,363	5,336	6,051	5,553	5,674	6,079	6,347
Clustering-based subsystems	18,051	17,585	19,425	19,647	19,055	19,441	20,434	19,860
Cofactors, Vitamins, Prosthetic Groups, Pigments	8,497	7,675	8,188	8,606	8,142	8,227	8,582	8,001
DNA Metabolism	5,461	5,331	5,191	5,559	5,321	5,717	5,824	5,855
Fatty Acids and Lipids	2,165	1,919	1,883	1,891	1,955	2,025	1,960	1,934
Macromolecular Synthesis	148	147	287	163	213	151	136	109
Membrane Transport	2,764	2,322	2,839	2,375	2,606	2,507	2,234	2,234
Metabolism of Aromatic Compounds	1,817	1,357	1,473	1,527	1,632	1,409	1,629	1,489
Miscellaneous	381	367	448	423	417	446	454	393
Motility and Chemotaxis	1,034	994	879	1,227	977	1,203	1,311	1,348
Nitrogen Metabolism	668	688	587	574	747	718	628	660
Nucleosides and Nucleotides	5,152	4,820	4,701	4,578	4,836	4,752	4,639	4,706
Phosphorus Metabolism	1,796	1,706	1,747	1,926	1,832	1,958	2,085	1,879
Photosynthesis	212	4,373	160	1,489	127	197	270	203
Potassium metabolism	648	591	586	631	620	755	838	817
Protein Metabolism	11,912	11,717	11,254	11,534	11,473	11,597	11,210	11,715
RNA Metabolism	5,133	4,889	4,660	4,813	4,811	4,744	5,068	4,981
Regulation and Cell signaling	1,196	1,127	1,400	966	1,356	1,360	1,076	1,056
Respiration	5,298	8,480	5,455	5,570	5,432	5,579	4,926	4,994
Secondary Metabolism	116	124	63	87	93	83	86	83
Stress Response	2,497	2,133	2,338	2,419	2,306	2,524	2,508	2,605
Sulfur Metabolism	1,604	1,354	1,673	1,430	1,446	1,240	1,320	1,317
Unclassified	6,235	5,677	6,567	5,763	6,672	6,019	5,555	5,794
Virulence	4,686	4,733	4,711	5,521	4,989	5,929	6,684	6,467

**Table 8:** Number of transcripts associated with the general SEED functional categories

**Subsystem Hierarchy 1**	**Jan 3:30pm**	**Jan 7pm**	**April 4pm**	**April 10pm**	**Aug 27 4pm**	**Aug 27 10pm**	**Aug 28 4am**
Amino Acids and Derivatives	261	536	204	198	21	144	443
Carbohydrates	886	1767	546	1302	530	1381	1256
Cell Division and Cell Cycle	83	191	52	63	96	56	80
Cell Wall and Capsule	154	353	317	297	153	113	221
Clustering-based subsystems	641	657	294	451	111	157	427
Cofactors, Vitamins, Prosthetic Groups, Pigments	215	457	130	248	24	13	469
DNA Metabolism	102	108	83	122	24	26	85
Fatty Acids and Lipids	84	28	17	27	0	28	10
Macromolecular Synthesis	0	0	5	2	2	0	0
Membrane Transport	44	19	237	83	2673	13	440
Metabolism of Aromatic Compounds	47	6	16	4	0	24	14
Miscellaneous	53	80	54	55	672	43	75
Motility and Chemotaxis	40	10	438	58	3	8	180
Nitrogen Metabolism	11	0	0	2	9	8	3
Nucleosides and Nucleotides	144	87	42	48	4	13	56
Phosphorus Metabolism	79	83	64	94	25	18	31
Photosynthesis	67	0	17	2	0	1	0
Potassium metabolism	29	13	3	13	4	2	7
Protein Metabolism	439	95	129	625	81	112	172
RNA Metabolism	1631	160	1813	702	907	2883	874
Regulation and Cell signaling	65	136	16	354	30	18	41
Respiration	174	20	26	97	125	31	109
Secondary Metabolism	18	3	1	0	0	0	1
Stress Response	100	175	42	229	5	43	56
Sulfur Metabolism	42	18	19	14	13	11	40
Unclassified	346	58	957	101	10	110	271
Virulence	152	847	385	716	385	651	546

## Highlights from the metagenome sequences

In general, in the samples, metagenomes were more similar than metatranscriptomes. Photosynthesis genes showed both seasonal and diel changes: specifically, 10 times greater photosynthetic potential in winter than in summer and greater abundance at night in January and April. Gene fragments annotated to proteorhodopsin showed virtually no seasonal or diel fluctuations, however: only approximately 0.07% of the annotated functional profile from each sample. Other seasonal differences in metagenomic profiles included a considerably higher winter abundance (compared to spring or summer) of archaeal genes associated with lipid synthesis, thermosome chaperonins, RNA polymerase, small subunit ribosomal proteins, DNA replication, and rRNA modification. Diel differences were apparent among genes involved in respiratory metabolism, which were more abundant at night.

The metatranscriptomic photosynthetic profiles were similar to those of the metagenomes in that photosynthesis genes were most abundant in January and virtually absent in August. Photosynthetic transcripts also were most abundant during the winter. On the other hand, unlike metagenomes, they were most abundant in the daytime in all months. Other seasonal differences in metatranscriptomic seasonal profiles included a greater abundance of transcripts related to membrane transport, especially amino acid transport, in summer when nutrients and dissolved organic material (DOM) are least abundant. The diel metatranscriptional profiles for January showed considerable difference in functions (in addition to photosynthesis); for example, transcripts relating to nitrogen cycling were most abundant during the day and were associated mainly with ammonification. Cell wall and capsule and cell division and cycle were upregulated at night, suggesting a nocturnal increase in cell division, potentially associated with the Cyanobacteria. Similarly, April samples showed a considerable up-regulation in RNA metabolism during the day, resulting primarily from an increase in group I intron and RNA polymerase transcripts. In August, transcripts with homology to membrane transport were upregulated during the day, while transcripts associated with motility and chemotaxis and with the synthesis of cofactors, vitamins, prosthetic groups, and pigments were considerably upregulated at night, suggesting that nocturnal motility and cellular activity (nucleotide and amino acid synthesis) were also upregulated.
